# Redesigning walking brochures using behaviour change theory: implications for walking intentions in natural environments

**DOI:** 10.1093/heapro/daaa150

**Published:** 2020-12-27

**Authors:** Lewis R Elliott, Mathew P White, Lora E Fleming, Charles Abraham, Adrian H Taylor

**Affiliations:** 1 European Centre for Environment and Human Health, University of Exeter Medical School, University of Exeter, c/o Knowledge Spa, RCHT, Truro, Cornwall TR1 3HD, UK; 2 Urban & Environmental Psychology Group, University of Vienna, Austria; 3 School of Psychological Sciences, Rm. 701, Redmond Barry Building, University of Melbourne, Parkville, VIC 3010, Australia; 4 Faculty of Medicine & Dentistry, University of Plymouth, N6, ITTC, Tamar Science Park, Plymouth, Devon PL6 8BX, UK

**Keywords:** physical activity, health communication, reasoned action, greenspace

## Abstract

Natural environments can be used to promote health through facilitating recreational walking. However, efforts to encourage this often neglect messages identified in psychological research that are effective at influencing intentions to walk. This is despite the National Institute for Health and Care Excellence stating that promotional efforts should utilize theoretical frameworks of behaviour change and be targeted towards less active adults. As an illustrative example, this experiment compared a prototypical recreational walking brochure with an “enhanced” version including such persuasive messages on people’s intentions to walk for recreation in natural environments. The enhanced brochure heightened intentions for inexperienced recreational walkers through our hypothesized mechanisms, but appeared to dissuade already-experienced walkers. Optimal messaging strategies in recreational walking brochures require tailoring to more and less active readerships. Guidelines are provided for authors of recreational walking brochures, though the principles and techniques could easily be extended to other means of outdoor walking promotion.

## INTRODUCTION

### Physical activity, natural environments and health and wellbeing

Physical inactivity is a key public health challenge, contributing to non-communicable diseases and premature mortality (Guthold *et al.*, 2018) with substantial economic costs (Scarborough *et al.*, 2011). There is therefore an urgent need for strategies which tackle physical inactivity at the community- or population-level (Baker *et al.*, 2015). Across all age groups, walking is one of the most important contributors to health-enhancing physical activity (Bélanger *et al.*, 2011) and is therefore seen as a manageable way for most people to increase their physical activity (Ogilvie *et al.*, 2007) as well as a public health priority (National Institute for Health and Care Excellence, 2012). Walking, even independently of other physical activity, has been associated with reduced risks of cardiovascular disease (Hamer and Chida, 2008) and reduced symptoms of depression (Robertson *et al.*, 2012). Despite its widespread accessibility, popularity, and substantial health benefits, the success of traditional interventions to promote increased walking is mixed (Foster *et al.*, 2011). Research has therefore shifted towards place-based approaches to support physical activity at a community- or population-level (Van Holle *et al.*, 2012).

The use of natural environments such as green spaces (e.g. parks, woods) or blue spaces (e.g. rivers, coastline) for recreational walking is one such place-based strategy. Natural environments support brisk levels of walking (Sellers *et al.*, 2012), and provide various landscapes for health-enhancing energy expenditure (Elliott *et al.*, 2015). They also elicit more positive affective responses compared with walking in more urbanized environments (Thompson Coon *et al.*, 2011), which may be particularly important for sustained physical activity behaviour (Rhodes and Kates, 2015). There is also a large body of evidence that suggests those with greater availability of green (Coombes *et al.*, 2010) and blue (White *et al.*, 2014) space tend to achieve higher levels of physical activity. Finally, there are financial benefits, with recreational physical activity in natural environments worth an estimated £2.2 billion in cost savings to health in England alone (White *et al.*, 2016).

### Promoting physical activity behaviour change in natural environments

Natural environments, therefore, appear to be a promising setting for promoting health-enhancing physical activity, in particular recreational walking, which may be sustainable in the longer-term. However, interventions to promote physical activity in natural environments have had limited success (Hunter *et al.* 2015). Contemporary approaches to supporting physical activity behaviour change focus on the complex socio-ecological systems which influence health (Sallis and Owen, 2015; Keshavarz Mohammadi, 2019). In contrast to the linear processes which underlie theoretical models of individual-level health behaviour change, these models embrace policy-level decisions, environmental change, behavioural settings, and their likely recurring feedback loops as key influencers of physical activity behaviour. This is especially true of the proliferation of ecological and planetary models of public health (Gagné and Lapalme, 2019). Despite such complexity, it is still recognized that individuals are at core of such models; their characteristics and motivations having the ability to alter processes in the ‘system’ (Sniehotta *et al.*, 2017).

Regarding recreational walking in natural environments, there is policy-level precedent for its promotion. The National Institute for Health and Care Excellence (NICE) encourage local authority directors for countryside management, the environment, parks, public health, and leisure services, to collaborate to ‘develop walking programmes for adults who are not active enough, based on an accepted theoretical framework for behaviour change’, and ‘ensure groups that are likely to be the least active are encouraged to participate, by addressing issues that may act as a barrier’ (National Institute for Health and Care Excellence, 2012, p. 18). Thus, even policy-level approaches acknowledge the necessity of understanding individual cognitions and actions when developing community-wide approaches to physical activity promotion. Despite this recommendation though, a content analysis of behaviour change messages in recreational walking brochures produced by such authorities in the UK concluded that their text frequently does not target theory-based behaviour change mechanisms known to influence physical activity uptake (Elliott *et al.*, 2016) and thus may not promote recreational walking optimally for less active adults. Although brochures or leaflets, even if effectively optimized, do not represent a solution on their own, they are a commonly used way to communicate the appeal of an area and walking opportunities.

Although paper or digital brochures are commonly used in interventions (Hunter *et al.*, 2015), they give little regard to tailoring messages to individual needs and readiness to engage in recreational walking in natural environments (Roberts *et al.*, 2016). The possibility therefore exists that accessing recreational walking brochures demotivates less active adults from recreational walking in natural environments due to assumptions about those reading them, potentially exacerbating inequalities in recreational walking (Dahmann *et al.*, 2010; Rind and Jones, 2011). Their increasing popularity as either central or adjunct means of physical activity promotion in exercise prescriptions (McKay *et al.*, 2009) and ‘green prescriptions’ (Van den Berg, 2017), means that it is crucial that messages in recreational walking brochures adhere to the NICE guidelines above. Basing the design of promotional materials on health behaviour change theory is not a new idea (Bandura, 1977; Carver and Scheier, 1982) but is imperative for transparency and understanding of how this material produces changes in behaviour (Abraham and Michie, 2008).

One way of helping local authorities produce theory-informed messages is through providing guidance on which persuasive messages are effective at encouraging less active adults to form stronger intentions to undertake recreational walking. These messages should target mechanisms (processes by which behaviour change occurs) and corresponding techniques (ways of affecting mechanisms) proposed by psychological theories (Abraham *et al.*, 2007; Gainforth *et al.*, 2011). Messages should be tailored to how motivated people are to change their recreational walking behaviour because the psychological change mechanisms that underlie the adoption of physical activity are different from those that underlie the maintenance of physical activity (Sniehotta *et al.*, 2005). For example, the theories of reasoned action and planned behaviour (Ajzen, 1991; Fishbein, 2008) have been used to describe how the change mechanisms of changing attitudes, raising normative beliefs and heightening perceived behavioural control can transition less active adults from physical activity motivation to volition (Courneya *et al.*, 2001), but adults attempting to maintain physical activity behaviours may require messages which target self-regulation processes (e.g. continuous self-monitoring of behaviour; Fjeldsoe *et al.*, 2011).

### This study

This study therefore hypothesized that ‘enhancing’ a recreational walking brochure with messages targeting attitudes, normative beliefs and perceived behavioural control could encourage so-called ‘non-walkers’ to form stronger intentions for recreational walking in natural environments compared with an existing brochure. We further hypothesized that such enhancements would *not* have comparable effects for people who already regularly undertook recreational walking. Ultimately, we aimed to provide guidance to local authority directors on how simple modifications could be made to existing recreational walking brochures (and by extension, potentially similar promotional materials) in order to adhere to NICE guidelines and thus more optimally promote recreational walking in natural environments for those who would usually be less likely to do this.

## MATERIALS AND METHODS

### Sample

Participants of the Cint panel (https://www.cint.com/consumer-insights-network/) were invited by email in September 2015 to participate. Cint participants earn small financial rewards for completing online surveys. Participants who exhibit systematic responses biases are removed (Meade and Craig, 2012), and precautions minimize the likelihood that surveys are automatically completed by machines. While socially desirable responses are possible (Behrend *et al.*, 2011), web-based recruitment methods typically attract diverse demographics (Gosling *et al.*, 2004). Although not a representative sample, participants were recruited from across the breadth of the UK.

### Experimental conditions

**Fig. 1:**
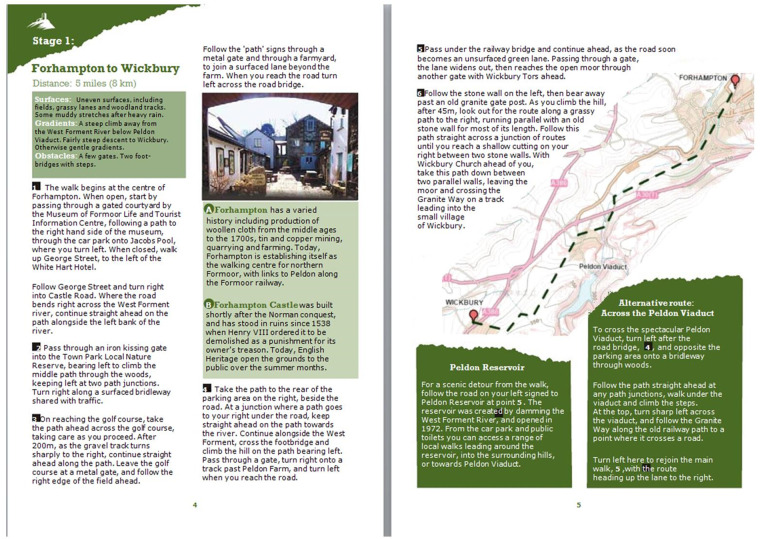
The original brochure.

**Fig. 2:**
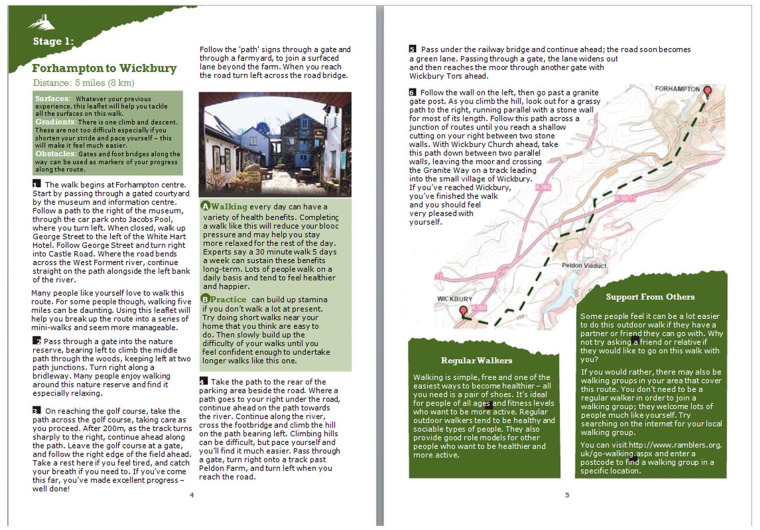
The ‘enhanced’ brochure.

A two-page extract from an existing recreational walking brochure from Devon, UK (Elliott *et al.*, 2016) was used as the ‘original brochure’ condition (Fig. 1). It described a riverside walk between two villages. Place names were fictionalized to reduce potential familiarity with the route; and a copyrighted map was replaced with an equivalent produced by Edina Digimap. The ‘enhanced brochure’ was kept as similar to the original brochure as possible, with only elements of the text being altered (Fig. 2). The following steps were taken to redesign the text of the enhanced walking brochure:

1. A content analysis of the original brochure was performed using a coding scheme (Elliott *et al.*, 2016) which identified potentially persuasive messages in recreational walking brochures (and their corresponding behaviour change techniques and psychological change mechanisms).
2. Text was omitted which was unable to be ascribed a persuasive message category according to the above coding scheme.
3. Repetitive text was also omitted (i.e. other messages in the brochure already targeted the corresponding behaviour change technique/mechanisms multiple times).
4. Guidance on behaviour change techniques that can be incorporated into written materials was consulted (Abraham and Kools, 2011). Potential techniques were selected if they targeted the psychological change mechanisms of changing attitudes, raising normative beliefs or heightening perceived behavioural control, as these have been shown to transition people from motivation to volition previously (Courneya *et al.*, 2001).
5. Messages operationalizing these techniques were written into the enhanced brochure being mindful not to interrupt the route directions which constituted the main narrative of the brochure. Supplementary Table SA displays a table of the change mechanisms and behaviour change techniques that were selected, together with the persuasive messages which were written into the ‘enhanced’ brochure to target these techniques and mechanisms.
6. Piloting these brochures helped clarify messages targeting injunctive normative beliefs (e.g. ‘your friends and family would support you completing this walk’), as too artificial. Such messages were deleted.

### Measures

Supplementary Table SB contains the full wording, response options and internal consistency coefficients (where applicable) pertaining to the measures described in the sections below.

#### Outcome variables

Recreational walking intentions were operationalized in two ways. The primary outcome analysed was a binary response to whether or not a participant requested further walking information about outdoor recreational walking in natural environments at the end of the survey. Requesting further information was interpreted as reflective of greater intentions to engage in such walks in the future. Two 7-point Likert-scale items measuring behavioural intentions (Ajzen, 2006) were collapsed as a secondary outcome variable. We refer to these two variables as ‘revealed intentions’ (more proximal to actual behaviour) and ‘stated intentions’ (more distal from actual behaviour), respectively (Ben-Akiva *et al.*, 1994).

#### Recreational walking status

To distinguish people who do not regularly undertake recreational walks in natural environments from those who do, an item which classified participants into five stages of readiness to change their recreational walking behaviour (Prochaska and Velicer, 1997) was created. Responses to the former three response options (reflecting amotivation or contemplation about changing behaviour in the long or short term) categorized participants as ‘non-walkers’ and responses to the latter two options (reflecting recent changes to behaviour, or stable behaviour patterns) categorized participants as ‘walkers’. Similar measures have good construct validity for exercise adoption (Cardinal, 1997).

#### Mediators

Items were created to assess whether the enhanced brochure impacted ‘non-walkers’ intentions through the proposed psychological change mechanisms of changing attitudes, raising normative beliefs, and heightening perceived behavioural control (Ajzen, 2006). Items measuring affective attitudes, instrumental attitudes, normative beliefs and perceived behavioural control were separately collapsed due to their high internal consistency (Supplementary Table SB). Collapsed instrumental and affective attitude items were further combined into a single ‘attitudes’ construct for the same reason (*α *= 0.89).

#### Covariates

The experiment also collected a series of demographic details which were operationalized in analyses as follows: sex (male, female), age (18–34, 35–48, 49–65), ethnicity (White-British, all other ethnicities), long-standing illness (yes, no) and annual pre-tax household income (five quintiles or ‘don’t know’). Ethnicity (Office for National Statistics, 2016), long-standing illness (Office for National Statistics, 2001), and income (Office for National Statistics, 2013) were collected according to national norms. These covariates have been independently shown to predict physical activity intentions or their antecedents (Wilson *et al.*, 2004; Ziegelmann et al., 2006; Kosma *et al.*, 2007; Amireault *et al.*, 2008; Gavin *et al.*, 2011). Measures adapted from a national survey (Natural England, 2019) queried the participant’s short- and long-term propensity for visiting natural environments as this has been shown to affect physical activity more generally (Coombes *et al.*, 2010) and therefore could affect intentions to be physically active (Calogiuri and Chroni, 2014).

### Procedure

Participants were randomized to one of the conditions based on a hyperlink sent to them in an invitation email. After giving consent, participants responded to questions concerning recreational walking status, short- and long-term propensity for visiting natural environments, ethnicity, long-standing illness and income on successive pages. Prior to seeing the brochure, they read text that was adapted from a study concerning immersion in natural environments (Weinstein *et al.*, 2009) in order to engage them with the task. They then could take as much as time as needed to read either the original or enhanced brochure in a new browser window.

Following this, they were asked whether they had read the brochure extract fully with those that did not being redirected to a debriefing page. Those that had proceeded to answer questions concerning the attitudes, descriptive norms, perceived behaviour control, and stated intentions. After this, they could also enter free responses as to what, if anything, had changed their motivation for recreational walking. Last, they responded to the item concerning revealed intentions. Supplementary Text SA contains a transcript of the full experiment.

### Analysis

Piloting suggested the brochure took a minimum of two minutes to read, so participants completing the experiment in <3 min were excluded *a priori*. Following guidance, participants were also excluded if they took one standard deviation longer than the mean completion time (Malhotra, 2008).

Logistic and linear regression models were constructed to analyse the impact of the brochures on revealed and stated intentions, respectively. The original brochure was used as a reference category as it is analogous to a ‘usual care’ condition in behavioural interventions (Freedland *et al.*, 2011). Models controlled for covariates and recreational walking status. Second, an interaction term was added between the experimental condition and recreational walking status to determine whether effects were stronger for ‘non-walkers’. Consistent with theories of reasoned action and planned behaviour, subsidiary mediation models tested whether differences in responses to the attitude, descriptive norm, and perceived behavioural control items mediated the effect of brochure condition on the two intention outcomes for ‘non-walkers’ (i.e. whether the enhanced brochure worked through the psychological change mechanisms we targeted).

Analyses were conducted in R v3.4.0 (R Core Team, 2018) using the ‘lavaan’ package (Rosseel, 2012).

## RESULTS

Originally, 535 participants were randomized to the two conditions (original *n *=* *269; enhanced *n *=* *266). Participants who indicated that they had not read the leaflet (*n* = 22), completed the experiment in under three minutes (*n* = 96) or over 20.26 min (*n* = 18), or had missing data (*n* = 4) were excluded. This left a total of *n* = 395, with 202 (51%) in the original brochure condition and 193 (49%) in the enhanced brochure condition. Females comprised 54% of the sample and the mean age was 42. ‘Non-walkers’ comprised 46% of the sample.

Participants did not differ between experimental conditions in terms of age [*F*(1, 393) = 0.00, *p* = 0.99, *η_p_* = 0.00], sex [*X*^2^ (2) = 0.17, *p* = 0.68], ethnicity [*X*^2^ (2) = 0.00, *p* = 0.95], household income [*X*^2^ (5) = 3.59, *p* = 0.61], illness/disability [*X*^2^ (1) = 0.13, *p* = 0.72] or propensity for visiting natural environments in the short term [*F* (1, 393) = 0.00, *p* = 0.99, *η_p_* = 0.00], or long term [*X*^2^ (7) = 6.21, *p* = 0.52]. Recreational walking status also did not differ with experimental condition [*X*^2^ (1) = 0.85, *p* = 0.36]. Descriptive statistics for the outcomes and mediators as a function of recreational walking status can be seen in Table 1. Of note, measures of dispersion were generally higher among ‘non-walkers’, potentially signifying more individual differences within this subgroup.

**Table 1:** Descriptive statistics for the two outcome variables and three proposed mediator variables

		Overall (*n* = 395)		Non-walkers (*n* = 182)		Walkers (*n* = 213)	
		Original brochure (*n* = 202)	Enhanced brochure (*n* = 193)	Original brochure (*n* = 88)	Enhanced brochure (*n* = 94)	Original brochure (*n* = 114)	Enhanced brochure (*n* = 99)
Revealed intentions	%	43.56	38.86	21.59	42.55	60.53	35.35
	SE	3.49	3.51	4.39	5.10	4.58	4.80
Stated intentions	*M*	4.99	5.32	4.20	4.78	5.60	5.83
	*SD*	1.77	1.54	1.69	1.63	1.59	1.25
Attitudes	*M*	5.27	5.51	4.88	5.20	5.58	5.80
	SD	1.18	1.27	1.22	1.38	1.05	1.09
Normative beliefs	*M*	5.16	5.32	4.68	4.97	5.53	5.65
	SD	1.39	1.46	1.38	1.52	1.30	1.32
Perceived behavioural control	*M*	4.59	5.30	4.33	4.85	5.43	5.73
	SD	1.52	1.47	1.65	1.64	1.21	1.15

Mean self-reported intention scores represent the average of two 7-point rating scales which were recoded: 1 = strongly disagree and 7 = strongly agree. Mean attitude score comprised the average score of four 7-point attitudinal items. Mean descriptive norm score and mean self-efficacy score comprised the average of two 7-point items each. Recreational walking status was dichotomized into two groups representing those who self-reported being in the precontemplation, contemplation and preparation stages of change (‘non-walkers’), and those who self-reported being in the action and maintenance stages of change (‘walkers’).

### Did the enhanced brochure strengthen recreational walking intentions overall?

Analysing all participants and controlling for potential confounds, the enhanced brochure did *not* prompt more requests for further recreational walking information than the original brochure [odds ratio (OR)* *=* *0.82; 95% CI: 0.54, 1.25; Supplementary Table SC), but did prompt stronger *stated* intentions (*b *=* *0.32; 95% CI: 0.03, 0.62). As expected, people classified as ‘walkers’ reported stronger revealed (OR* *=* *2.10; 95% CI: 1.33, 3.34) and stated (*b *=* *1.03; 95% CI: 0.71, 1.35) intentions than ‘non-walkers’ overall.

### Were these effects stronger for ‘non-walkers’?

**Fig. 3:**
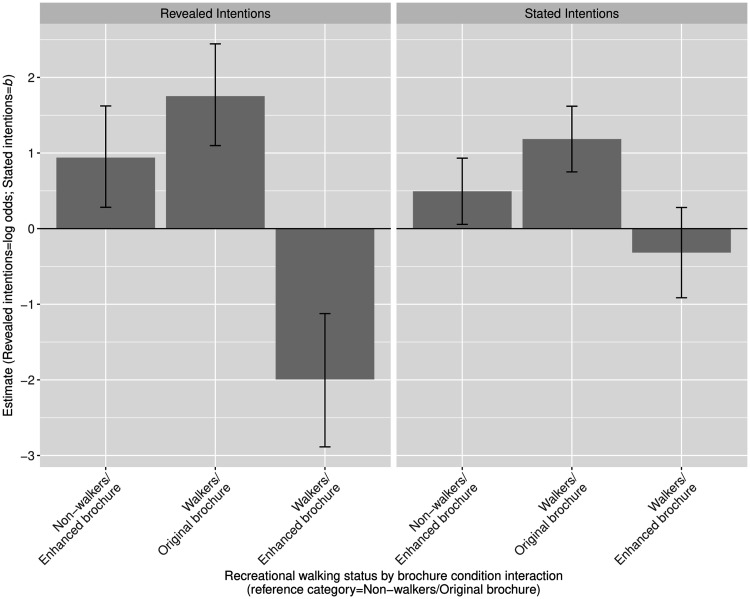
The effect of the interaction between the experimental condition and recreational walking status on both outcome variables.

After adding an interaction term between the experimental brochure condition and recreational walking status, distinct patterns for ‘non-walkers’ and ‘walkers’ emerged in terms of both outcome variables (Fig. 3). First, supporting our hypotheses, ‘non-walkers’ who read the enhanced brochure made significantly more requests for recreational walking information than ‘non-walkers’ who read the original brochure (OR* *=* *2.56; 95% CI: 1.33, 5.07; Supplementary Table SC). Second, ‘walkers’ who read the original brochure made significantly more requests than ‘non-walkers’ who read the original brochure (OR* *=* *5.77; 95% CI: 3.00, 11.51). Last, and unexpectedly, ‘walkers’ who read the enhanced brochure made significantly *fewer* requests than ‘non-walkers’ who read the original brochure (OR* *=* *0.14; 95% CI: 0.06, 0.33). The pattern was the same for stated intentions, though effects were slightly weaker, and in the case of the latter unexpected finding, not significant (Fig. 3 and Supplementary Table SC). Associations between the other potential confounds and the outcome variables remained broadly consistent after the addition of this interaction term.

These analyses were repeated including participants with atypically short or long completion times (see ‘Analysis’ section) and all effects were weaker (Supplementary Table SC), justifying our decision to exclude on this basis.

### Did differences in attitudes, descriptive norms and perceived behavioural control mediate the effect of the brochures on recreational walking intentions for ‘non-walkers’?

**Fig. 4:**
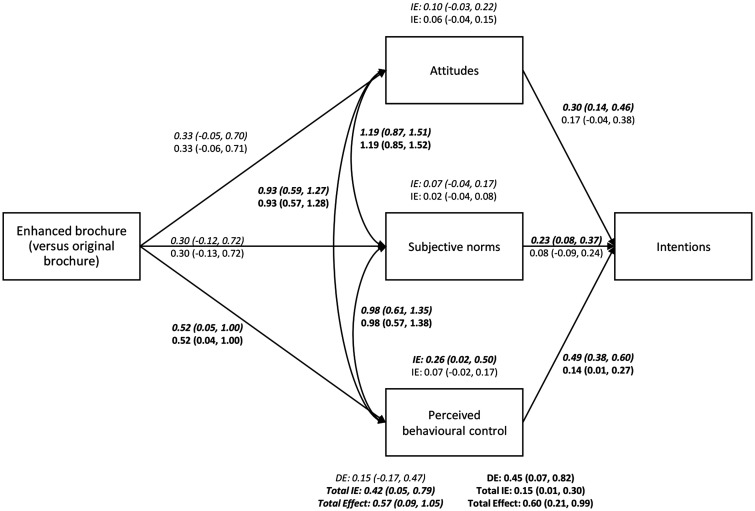
Mediation models demonstrating the effect of reading the enhanced brochure (vs. original brochure) on revealed intentions and stated intentions (in italics) through attitudes, subjective norms, and perceived behavioural control for ‘non-walkers’. Significant effects and covariances are highlighted in bold. Estimates and 95% CIs are presented. DE, direct effect; IE, indirect effect; NB: For revealed intentions, the diagonally-weighted least squares estimator with probit link function was used (Rosseel, 2012); hence, the estimates cannot be compared with ORs or log odds. For stated intentions, the maximum likelihood estimator was used. Slightly different CIs for covariances and regressions of the mediators on the experimental brochure condition are a consequence of the number of iterations of the model before successful convergence.

As coefficients did not change substantially following the inclusion of covariates, the mediation models excluded covariates in favour of just the experimental conditions, outcomes, and mediators. For ‘non-walkers’, perceived behavioural control significantly mediated the effects of the enhanced brochure on stated intentions (*b *=* *0.26; 95% CI: 0.02, 0.50), explaining ≈45% of the variance in the total effect; but attitudes and subjective norms did not mediate the effect (Fig. 4). The combination of all three constructs also mediated the effects of the enhanced brochure on stated intentions for ‘non-walkers’ (*b *=* *0.42; 95% CI: 0.05, 0.79), explaining ≈73% of the variance in the total effect.

None of the three constructs significantly mediated the effects of the enhanced brochure on the revealed intentions for ‘non-walkers’ on their own; but the combination of all three did (*b *=* *0.15; 95% CI: 0.01, 0.30), explaining ≈25% of the variance in the total effect. As a comparison, the same models were performed for ‘walkers’ (Supplementary Fig. SA) and as predicted, neither differences in attitudes, descriptive norms, perceived behavioural control, nor their sum, mediated the relationship between the experimental brochure condition and either outcome variable.

## DISCUSSION

In order to maximize the potential that natural environments have for encouraging recreational walking, such experiences need to be optimally promoted, especially to less active people. This experiment compared an archetypal walking brochure with one which had been ‘enhanced’ using persuasive messages which targeted theory-based psychological change mechanisms. As hypothesized, this enhanced brochure prompted stronger recreational walking intentions among ‘non-walkers’—they made over twice as many requests for further walking information and on average reported intentions half a point higher compared with reading the original brochure. Conversely, ‘walkers’ who read the enhanced brochure were much *less* likely to request further walking information than ‘walkers’ who read the original brochure. We also demonstrated that differences in the three psychological change mechanisms targeted were responsible for influencing the intentions of ‘non-walkers’, (especially perceived behavioural control), but not ‘walkers’. This study further justifies the need for behaviour change theory when designing recreational walking brochures, and indeed physical activity interventions more generally (Rhodes *et al.*, 2019). However, this study also demonstrates that brochure authors (or intervention designers) need to be flexible with their approach to selecting theories (Peters and Crutzen, 2017), as the kinds of persuasive messages (and underlying behaviour change techniques) that successfully strengthen intentions for one audience, may not work for a different audience.

### Implications for the creation of outdoor recreational walking brochures for ‘non-walkers’

The main implication of these findings is that two distinct types of outdoor recreational walking brochure could be developed to heighten outdoor walking intentions among two target audiences. The first of these are ‘non-walkers’, i.e. those who have not contemplated recreational walking in natural environments, or those that have contemplated this, but have currently failed to act on these thoughts. Consistent with previous research (Courneya *et al.*, 2001), this study suggests that as well as route instructions, adding text to brochures which attempts to change people’s attitudes towards outdoor recreational walking, promote normative beliefs about what similar others may do, or raise confidence for such walking, may help ‘non-walkers’ form stronger intentions to walk in natural environments by encouraging them to contemplate further how to undertake such action.

Brochure designers can consider influencing both instrumental attitudes (advantages of undertaking outdoor recreational walking) and affective attitudes (emotions stimulated by performing outdoor recreational walking). This study cannot deconstruct which type of message may be more persuasive, but studies have previously suggested that affective attitudes may be more important for predicting the uptake of physical activity (Lowe *et al.*, 2002; French *et al.*, 2005). Brochure designers also have the opportunity to describe the outdoor recreational walking behaviour of peers or encourage recipients to seek social comparison opportunities (e.g. encouraging people to interact with others in walking groups; Supplementary Table SA). However, normative beliefs are typically weak predictors of physical activity uptake (Downs and Hausenblas, 2005) which may explain their weaker influence in our mediation models.

There are multiple ways in which brochure designers can promote perceived behavioural control (i.e. raising people’s confidence for performing recreational walking in natural environments). In this study, we targeted this change mechanism in a number of ways (Supplementary Table SA): (i) prompting reattribution of past failures (e.g. past failed attempts to start outdoor recreational walking); (ii) prompting barrier identification and planning in relation to anticipated barriers (e.g. difficulty in climbing hills); (iii) setting graded tasks/goals (e.g. prompting practice of multiple, shorter walks); (iv) providing feedback on performance (e.g. commending the recipient on successful completion of a stage); (v) using arguments to bolster confidence (e.g. arguing against self-doubt and asserting that they can succeed in changing their behaviour); and (vi) prompting organization of social support (e.g. joining a walking group). This change mechanism (enhancing confidence) may be prompted by a variety of behaviour change techniques (Abraham and Kools, 2011) and was the most frequently targeted in the enhanced brochure, potentially explaining why it was the most important construct in relation to predicting behavioural intentions.

Previous research has further demonstrated that confidence-building aspects of perceived behavioural control are particularly important for forming intentions to take up physical activity more generally (Hagger *et al.*, 2002). ‘Non-walkers’’ quotes about the enhanced brochure illustrated the persuasive nature of these messages: ‘*it is a very positive leaflet that made me feel comfortable in taking it on despite having no experience*’; and ‘*it was very encouraging and felt like it was addressing me as an individual and not just giving the route directions, which is the norm…it made me want to start walking again*’.

Although there were not sufficient responses to this open question to undertake systematic qualitative analysis, these quotes at least suggest that for some ‘non-walkers’, enhancing confidence for walking in natural environments was the primary means by which they formed stronger intentions, supporting the quantitative findings. All responses to this open question can be found in the raw data upon request from the authors.

Although this discussion suggests that designing persuasive messages targeting perceived behavioural control and attitudes (especially affective attitudes) may be most effective at encouraging ‘non-walkers’ to contemplate future outdoor recreational walking, our mediation models also suggest that the combination of our three key change mechanisms is also important. Brochure designers are encouraged to consult practical guidance (e.g. Abraham and Kools, 2011) on how to incorporate persuasive messages targeting these change mechanisms in a wider variety of ways which go beyond the techniques employed in this study.

### Implications for the creation of outdoor recreational walking brochures for ‘walkers’

Brochure designers may also wish to design persuasive messages for ‘walkers’ who are more familiar with walking trails. In this study, these were classified as people who were already undertaking outdoor recreational walking in natural environments. These people were substantially *less* likely to request further walking information after reading the enhanced brochure, suggesting that the changes to the original brochure actually *dissuaded* these individuals from walking in natural environments.

Although qualitative responses were too scarce to draw definitive conclusions, some responses revealed that the enhanced brochure may have lowered intentions for these ‘walkers’ because they found the language within to be patronizing: ‘*The tone of the leaflet was quite condescending*’, and ‘*leave the motivational stuff to a separate section…it is annoying and patronizing’*. This could be seen as analogous to the notion of ‘baby talk’ in health psychology research where a health care provider underestimates the patient’s knowledge and uses language perceived as patronizing, thus leading to disengagement (Waitzkin, 1985).

Generally speaking, the effects of both brochures on ‘walkers’’ intentions were the strongest observed effects in this study; greater than, e.g. the effects of sex, age, ethnicity or income on these intentions (Supplementary Table SC). Thus, ‘walkers’, even more so than ‘non-walkers’, could be particularly responsive to the written content of recreational walking brochures.

The original brochure may appeal more to ‘walkers’ because it already contained the sort of information that was more persuasive for this group (Elliott *et al.*, 2016). This could be messages which highlighted heritage features in natural environments, or signposted the reader to nearby amenities (McCormack *et al.*, 2010). Although these messages could be construed to be related to material consequences of recreational walking (and thus, could change attitudes towards the behaviour), we are unable to provide definitive guidance on what types of message may be most persuasive for ‘walkers’ because our mediation models did not identify differences in attitudes, normative beliefs or perceived behavioural control between the two brochures for this audience (Supplementary Fig. SA). However, we do recommend that intentionally designing theory-derived persuasive messages in recreational walking brochures for this group should not involve the use of text that could be construed as ‘patronizing’, regardless of the change mechanisms that are targeted.

Without further knowledge of the guidelines and management considerations that factor into how a brochure advertising recreational walking in natural environments is created and written, it is difficult to make recommendations on how different design guidelines could be implemented in reality. Nonetheless, this study provides evidence that the implementation of guidelines which encourage the use of evidence-based persuasive messages is effective at changing recreational walking intentions. Although only intentions were measured in this study, meta-analysis has shown that targeting the same mechanisms have small but significant effects on actual behaviour change (Webb and Sheeran, 2006). Furthermore such tailored media could support so-called ‘green prescriptions’, i.e. direct recommendations from health care professionals to spend more time in natural settings to improve health and wellbeing (Van den Berg, 2017).

### Limitations

First, while only the text component of the original brochure was manipulated in this study, there are numerous stylistic features that may aid or inhibit comprehension of the written text, e.g. graphical illustrations of specific behaviours (Kools *et al.*, 2006), or coloured tabs and pictorials (Kools *et al.*, 2007). Second, the brochures used in this experiment described a linear route in a semi-rural riverside location, but people’s preferences for features of recreational walking routes differ with their demographics (Davies *et al.*, 2012). Replications of this study with different audiences and different exemplar brochures are necessary to determine how generalizable the current findings are.

Third, the three psychological change mechanisms we targeted do not necessarily support maintenance of behaviour change (Kwasnicka *et al.*, 2016), i.e. the brochure does not propose an explanation as to how individuals could maintain physically active behaviours once they have initiated these behaviours. Future attempts to design persuasive messages in recreational walking brochures may wish to draw on other behavioural models, such as the model of behavioural maintenance (Rothman, 2000), in order to elicit more sustained changes in people’s walking behaviour.

We are also aware that specific text substitutions may have influenced our outcomes in unintended ways. For example, we changed the text *‘a steep climb… and a fairly steep descent’* to *‘there is one climb and descent. These are not too difficult if you shorten your stride and pace yourself—this will make it feel much easier’*, with additional text reading *‘climbing hills can be difficult, but pace yourself and you’ll find it much easier’.* Removing the word ‘steep’, notwithstanding other factors affecting perceived steepness (Schnall *et al.*, 2008, 2010; Taylor-Covill and Eves, 2016), may have affected intentions, or their antecedents, measured in this study. However, rather than misleading readers, we were simply acknowledging that individuals may find the terrain difficult and that by implementing a simple strategy this challenge could be overcome.

More generally, the generalizability of our results to other contexts is questionable. The materials used in our study may be culturally specific to a British population, and our analysis cannot address how likely less active populations are to access such materials. Future research could therefore focus on replication in populations with different cultures of walking and qualitative explorations of similar materials with target populations as part of their development. We also recognize that tailored communication messages are already ubiquitous in mobile health applications, but applications like these are typically geared towards populations already motivated to change their behaviour (Bardus *et al.*, 2016), and in any case we contend that there is still good evidence to suggest that greenspace interventions fail to make the best use of potentially persuasive physical activity behaviour change messages (Roberts *et al.*, 2016).

Last, our findings cannot be seen in isolation from the wider socio-ecological systems that influence physical activity (Sallis and Owen, 2015). If the ultimate public health goal is reducing physical inactivity, then policy-level initiatives such as improving accessibility or safety of walking settings may be most effective (Panter *et al.*, 2019). Nonetheless, intervening without understanding behavioural complexities and motivations of individuals would ignore a key part of these complex socio-ecological systems and potentially undermine interventions (Rhodes *et al.*, 2019), so explorations like those in this study remain worthwhile endeavours.

## CONCLUSION

To ensure natural environments are used for recreational walking, especially by people who are typically less active, these opportunities should be effectively promoted using appropriate persuasive messages. However, current materials may not do so optimally. This study found that enhancing existing materials with theory-based persuasive messaging was effective at strengthening walking intentions among less active adults. We demonstrated a need for two types of recreational walking brochure: (i) those appealing to ‘non-walkers’ which attempt to increase intentions to engage in outdoor recreational walking in natural environments by targeting determinants such as perceived behavioural control; and, (ii) those aimed at already-motivated ‘walkers’ which can assume motivation, avoid the use of patronizing language, and focus on extrinsic features of a recreational walking route with clear instructions, thus supporting walking maintenance. Brochure authors are encouraged to make use of these guidelines and other existing practical guidance on how to construct messages which target evidence-based antecedents of physical activity behaviour change and to be vigilant to the variability in effective communication strategies for different target audiences. Provision of supportive natural environments for physical activity is necessary, but it is not a sufficient means of altering community- or population-level physical activity behaviour. Individualized approaches, such as those presented in this article remain fundamental to altering physical activity behaviours.

## Supplementary Data

Supplementary material is available at *Health Promotion International* online.

## ETHICAL APPROVAL

This study was approved by the University of Exeter’s Sport and Health Sciences Research Ethics Committee.

## Supplementary Material

daaa150_Supplementary Materials
