# Supplementary material for: Redesigning walking brochures using behaviour change theory: implications for walking intentions in natural environments
Source: Health Promot Int. 2020 Dec 27;36(4):1126–39. doi: 10.1093/heapro/daaa150 (PMC8527999; doi:10.1093/heapro/daaa150)
Supplement: daaa150_Supplementary Materials [file daaa150_Supplementary_Materials.docx]

| Supplementary Table A. Persuasive messages added to the 'enhanced' brochure grouped by behaviour change techniques and associated change mechanisms derived from Abraham (2012)^1^. | | |
| --- | --- | --- |
| **Change mechanism (Abraham, 2012)^1^** | **Behaviour change technique (Abraham, 2012)^1^** | **Persuasive messages added to the 'enhanced' brochure** |
| Techniques designed to change feelings (or affective attitudes) associated with adopting or ceasing behaviours | Describe likely emotional (or affective) consequences of behaviour | "Many people enjoy walking around this nature reserve and find it especially relaxing." |
| Techniques designed to change beliefs about the benefits and costs of behaviour/s  – i.e., to change instrumental (or cognitive) attitudes. | Describe likely material consequences of behaviour | "Walking every day can have a variety of health benefits. Completing a walk like this will reduce your blood pressure and may help you stay more relaxed for the rest of the day. Experts say a 30 minute walk 5 days a week can sustain these benefits long-term." |
|  |  | "Walking is simple, free and one of the easiest ways to become healthier." |
| Techniques designed to enhance self-efficacy | Prompt reattribution of past successes and failures | "Whatever your previous experience, this leaflet will help you tackle all the surfaces on this walk." |
|  | Prompt barrier identification and planning in relation to anticipated barriers | "There is one climb and descent. These are not too difficult especially if you shorten your stride and pace yourself – this will make it feel much easier." |
|  |  | "Take a rest here if you feel tired, and catch your breath if you need to." |
|  |  | "If you would rather, there may also be walking groups in your area that cover this route. You don’t need to be a regular walker in order to join a walking group; they welcome lots of people much like yourself. Try searching on the internet for your local walking group." |
|  |  | "You can visit http://www.ramblers.org. uk/go-walking.aspx and enter a postcode to find a walking group in a specific location." |
|  | Set graded tasks/goals | "For some people though, walking five miles can be daunting. Using this leaflet will help you break up the route into a series of mini-walks and seem more manageable." |
|  |  | "Practice can build up stamina if you don’t walk a lot at present. Try doing short walks near your home that you think are easy to do. Then slowly build up the difficulty of your walks until you feel confident enough to undertake longer walks like this one." |
|  | Provide feedback on performance | "If you’ve come this far, you’ve made excellent progress – well done!" |
|  |  | "If you’ve reached Wickbury, you’ve finished the walk and you should feel very pleased with yourself." |
|  | Use argument to bolster self-efficacy | "Climbing hills can be difficult, but pace yourself and you’ll find it much easier." |
|  |  | "It’s ideal for people of all ages and fitness levels who want to be more active." |
| Techniques designed to change (normative) beliefs about other people’s behaviour and approval of recipients’ behaviour | Provide information about others’ behaviour | "Many people like yourself love to walk this route." |
|  |  | "Lots of people walk on a daily basis and tend to feel healthier and happier." |
|  |  | "Many people enjoy walking around this nature reserve and find it especially relaxing." |
|  |  | "It’s ideal for people of all ages and fitness levels who want to be more active." |
| Techniques designed to facilitate behaviour change by prompting environmental change^2^ | Teach to use environmental prompts/cues | "Gates and foot bridges along the way can be used as markers of your progress along the route." |
|  | Prompt organisation of social support | "Some people feel it can be a lot easier to do this outdoor walk if they have a partner or friend they can go with. Why not try asking a friend or relative if they would like to go on this walk with you?" |
|  |  | "If you would rather, there may also be walking groups in your area that cover this route. You don’t need to be a regular walker in order to join a walking group; they welcome lots of people much like yourself. Try searching on the internet for your local walking group." |
|  |  | "You can visit http://www.ramblers.org. uk/go-walking.aspx and enter a postcode to find a walking group in a specific location." |
| Techniques to designed foster a positive behaviour-related identity^3^ | Provide a positive group identity for those engaging in the target behaviour | "Regular outdoor walkers tend to be healthy and sociable types of people." |
|  | Prompt identification as role model/ position advocate | "They also provide good role models for other people who want to be healthier and more active." |
| N.B Some persuasive messages appear under more than one behaviour change technique or change mechanism; this is because persuasive messages are often not mutually exclusive in the mechanisms they work through.  ^1^ Abraham, C. (2012). Mapping change mechanisms onto behaviour change techniques: a systematic approach to promoting behaviour change through text. In C. Abraham & M. Kools (eds.) *Writing health communication. An evidence-based guide* (pp. 99-116). London, UK: Sage. Consult this book for full descriptions of each behaviour change technique.  ^2^ Whilst not explicitly referring to attitudinal, normative, or efficacy messages, the persuasive messages subsumed under this change mechanism can additionally be said to prompt barrier identification and planning (i.e. enhance self-efficacy).  ^3^ Whilst not explicitly referring to attitudinal, normative, or efficacy messages, the persuasive messages subsumed under this change mechanism can additionally be said to encourage recipients to seek social comparison opportunities (i.e. change normative beliefs; this is another behaviour change technique from Abraham, 2012). | | |

| Supplementary Table B. Measures used in the present study that pertain to the outcome variables, recreational walking status, and mediators. | | | |
| --- | --- | --- | --- |
| Construct | Item(s) | Response | Cronbach's Alpha |
| Recreational walking status | When we refer to “walking routes” we mean walking through trails/routes/paths for pleasure in outdoor natural environments such as open spaces in and around towns and cities, the coast and the countryside.  These could be short or long, circular or linear, easy or difficult, marked or unmarked, walking routes. We do not mean walking these routes for transport purposes (to get to and from places) or walking around your own private garden or land.  Please check the statement that most applies to yourself: | “I do not regularly complete walking routes and am not thinking about starting” “I do not regularly complete walking routes but I am thing about starting (but not in the next month)”  “I do not regularly complete walking routes but am thinking about starting in the next month”  “I do regularly complete walking routes and have done so for less than 6 months” “I do regularly complete walking routes and have done so for over 6 months”  *Respondent selects one option.* | n/a |
| Instrumental attitudes | “Doing this outdoor walking route would be…”  "Doing this outdoor walking route would be…” | *1 (bad) to 7 (good)\|  1 (very worthwhile) to 7 (not at all worthwhile)*^a^ | .78 |
| Affective attitudes | “Doing this outdoor walking route would be…”  “Doing this outdoor walking route would be…” | *1 (pleasant) to 7 (unpleasant)*^a^ *1 (exciting) to 7 (boring)*^a^ | .77 |
| Descriptive norms | “Other people like me complete walking routes like this.”  “A wide range of people complete walking routes like this.” | *1 (strongly agree) to 7 (strongly disagree)*^a^ *1 (strongly agree) to 7 (strongly disagree)*^a^ | .79 |
| Perceived behavioural control | “I am confident I could complete walking routes like this.”  “Completing walking routes like this would be…” | *1 (true) to 7 (false)*^a^ *1 (very difficult) to 7 (very easy)* | .76 |
| Stated intentions | “I would be willing to complete walking routes like this.”  “In the future, I intend to complete walking routes like this.” | *1 (strongly agree) to 7 (strongly disagree)*^a^ *1 (strongly agree) to 7 (strongly disagree)*^a^ | .90 |
| Revealed intentions | If you would like further information on walking in your area, please select “show me walking information and submit” below. If you do not wish to be shown this, please just select “take me to submission page.” | Show me walking information and submit  Take me to submission page  *Respondent selects one option.* | n/a |
| ^a^ These items were reverse coded in analysis.  Notes: Items are listed in the order they appeared in the experiment. Descriptive norms and efficacy items were generalized to read “similar walking routes” as opposed to the walking route the respondent had just read about or imagined because the enhanced brochure content was designed to raise normative beliefs and enhance efficacy for recreational walking in natural environments more generally. Affective and instrumental attitudes were, in general though not exclusively, more often tied to the walking route advertised. Despite this subtle difference in the target behaviour, it is likely that participant's responses were anchored on the walk they had just read about or imagined anyway (Tversky and Kahneman 1974). Recreational walking status was adapted from a previous stages of change question used for exercise adoption (Cardinal 1997). The theory of planned behaviour mediators and stated intentions items were adapted from guidance on creating a theory of planned behaviour questionnaire (Ajzen 2006). | | | |

| Supplementary Table C. Unadjusted and fully-adjusted odds ratios and regression coefficients, together with 95% confidence intervals, for models which predict revealed and stated intentions. Models both without and with an interaction between experimental brochure condition and recreational walking status are presented (hypotheses (a) and (b) respectively). A fully-adjusted model including all participants who are excluded from the main analysis due to atypically short or long questionnaire completion times is also presented for comparison. | | | | | | | | |
| --- | --- | --- | --- | --- | --- | --- | --- | --- |
|  | Models without interaction | | | | Models with interaction | | | |
|  | Revealed intentions | | Stated intentions | | Revealed intentions | | Stated intentions | |
| *Unadjusted (n=395)* | (Hosmer & Lemeshow=.02) (Nagelkerke=.04) | | (Adjusted R^2^=.14) | | (Hosmer & Lemeshow=.06) (Nagelkerke=.11) | | (Adjusted R^2^=.14) | |
|  | OR | CI | *b* | CI | OR | CI | *b* | CI |
| Intercept | 0.52 | (0.36, 0.75) | 4.30 | (4.02, 4.57) | 0.28 | (0.16, 0.45) | 4.20 | (3.88, 4.53) |
|  |  |  |  |  |  |  |  |  |
| *Brochure (original brochure=ref)* | - | - | - | - | - | - | - | - |
| Enhanced brochure | 0.85 | (0.56, 1.27) | 0.39 | (0.09, 0.70) | 2.69 | (1.41, 5.24) | 0.57 | (0.12, 1.02) |
| *Recreational walking status ("non-walkers"=ref)* | - | - | - | - | - | - | - | - |
| "Walkers" | 1.97 | (1.31, 2.99) | 1.23 | (0.92, 1.53) | 5.57 | (3.01, 10.68) | 1.39 | (0.96, 1.82) |
| Enhanced brochure x "Walkers" | - | - | - | - | 0.13 | (0.06, 0.31) | -0.34 | (-0.95, 0.28) |
|  |  |  |  |  |  |  |  |  |
| *Fully-adjusted (n=395)* | (Hosmer & Lemeshow=.05) (Nagelkerke=.09) | | (Adjusted R^2^=.20) | | (Hosmer & Lemeshow=.09) (Nagelkerke=.15) | | (Adjusted R^2^=.20) | |
|  | OR | CI | *b* | CI | OR | CI | *b* | CI |
| Intercept | 0.67 | (0.26, 1.70) | 3.68 | (3.01, 4.35) | 0.39 | (0.14, 1.04) | 3.60 | (2.92, 4.29) |
|  |  |  |  |  |  |  |  |  |
| *Brochure (original brochure=ref)* | - | - | - | - | - | - | - | - |
| Enhanced brochure | 0.82 | (0.54, 1.25) | 0.32 | (0.03, 0.62) | 2.56 | (1.33, 5.07) | 0.49 | (0.06, 0.93) |
| *Recreational walking status ("non-walkers"=ref)* | - | - | - | - | - | - | - | - |
| "Walkers" | 2.10 | (1.33, 3.34) | 1.03 | (0.71, 1.35) | 5.77 | (3.00, 11.51) | 1.18 | (0.75, 1.62) |
| Enhanced brochure x "Walkers" | - | - | - | - | 0.14 | (0.06, 0.33) | -0.32 | (-0.91, 0.28) |
|  |  |  |  |  |  |  |  |  |
| *Sex (male=ref)* | - | - | - | - | - | - | - | - |
| Female | 1.36 | (0.89, 2.08) | -0.09 | (-0.40, 0.21) | 1.26 | (0.81, 1.96) | -0.11 | (-0.41, 0.19) |
| *Age (18-34 year olds=ref)* | - | - | - | - | - | - | - | - |
| 35-48 year olds | 1.96 | (1.15, 3.39) | 0.00 | (-0.37, 0.38) | 1.96 | (1.13, 3.44) | -0.00 | (-0.38, 0.38) |
| 49-65 year olds | 1.60 | (0.94, 2.75) | -0.08 | (-0.46, 0.29) | 1.61 | (0.93, 2.81) | -0.09 | (-0.46, 0.29) |
| *Ethnicity (all other ethnicities=ref)* | - | - | - | - | - | - | - | - |
| White-British | 0.65 | (0.37, 1.17) | -0.16 | (-0.57, 0.25) | 0.61 | (0.34, 1.11) | -0.16 | (-0.58, 0.25) |
| *Long-standing illness/disability (yes=ref)* | - | - | - | - | - | - | - | - |
| No | 0.80 | (0.47, 1.35) | 0.65 | (0.28, 1.02) | 0.77 | (0.45, 1.33) | 0.65 | (0.27, 1.02) |
| *Income (less than £15,000=ref)* | - | - | - | - | - | - | - | - |
| £15,000-£25,000 | 0.45 | (0.21, 0.92) | 0.30 | (-0.20, 0.81) | 0.48 | (0.23, 1.01) | 0.32 | (-0.18, 0.82) |
| £25,000-£35,000 | 0.68 | (0.34, 1.35) | 0.13 | (-0.36, 0.62) | 0.73 | (0.36, 1.48) | 0.14 | (-0.34, 0.63) |
| £35,000-£50,000 | 0.67 | (0.32, 1.37) | 0.36 | (-0.16, 0.87) | 0.67 | (0.31, 1.40) | 0.36 | (-0.16, 0.87) |
| £50,000 and above | 0.88 | (0.44, 1.73) | 0.69 | (0.20, 1.18) | 0.86 | (0.43, 1.74) | 0.69 | (0.20, 1.17) |
| Don't know | 0.68 | (0.27, 1.69) | -0.47 | (-1.12, 0.18) | 0.73 | (0.28, 1.88) | -0.46 | (-1.11, 0.20) |
| *Long-term nature visit propensity (less frequent visitors=ref)* | - | - | - | - | - | - | - | - |
| More frequent visitors | 1.06 | (0.64, 1.73) | 0.21 | (-0.14, 0.56) | 1.06 | (0.63, 1.75) | 0.21 | (-0.14, 0.56) |
| *Short-term visit propensity* | 1.00 | (0.96, 1.05) | 0.03 | (-0.01, 0.06) | 1.00 | (0.96, 1.06) | 0.03 | (-0.01, 0.06) |
| *Fully-adjusted (n=509; including previously excluded participants)* | (Hosmer & Lemeshow=.04) (Nagelkerke=.07) | | (Adjusted R^2^=.17) | | (Hosmer & Lemeshow=.06) (Nagelkerke=.11) | | (Adjusted R^2^=.17) | |
|  | OR | CI | *b* | CI | OR | CI | *b* | CI |
| Intercept | 0.92 | (0.41, 2.09) | 3.50 | (2.89, 4.11) | 0.60 | (0.25, 1.42) | 3.46 | (2.83, 4.08) |
|  |  |  |  |  |  |  |  |  |
| *Brochure (original brochure=ref)* | - | - | - | - | - | - | - | - |
| Enhanced brochure | 0.75 | (0.52, 1.08) | 0.22 | (-0.05, 0.49) | 1.78 | (1.03, 3.14) | 0.32 | (-0.08, 0.72) |
| *Recreational walking status ("non-walkers"=ref)* | - | - | - | - | - | - | - | - |
| "Walkers" | 1.68 | (1.13, 2.50) | 0.97 | (0.68, 1.26) | 3.62 | (2.09, 6.38) | 1.06 | (0.66, 1.45) |
| Enhanced brochure x "Walkers" | - | - | - | - | 0.21 | (0.10, 0.44) | -0.18 | (-0.73, 0.36) |
|  |  |  |  |  |  |  |  |  |
| *Sex (male=ref)* | - | - | - | - | - | - | - | - |
| Female | 1.17 | (0.81, 1.70) | 0.07 | (-0.21, 0.34) | 1.12 | (0.76, 1.63) | 0.06 | (-0.21, 0.34) |
| *Age (18-34 year olds=ref)* | - | - | - | - | - | - | - | - |
| 35-48 year olds | 1.45 | (0.92, 2.30) | -0.07 | (-0.41, 0.26) | 1.48 | (0.93, 2.36) | -0.07 | (-0.41, 0.26) |
| 49-65 year olds | 1.42 | (0.90, 2.27) | 0.03 | (-0.31, 0.37) | 1.41 | (0.88, 2.26) | 0.03 | (-0.32, 0.37) |
| *Ethnicity (all other ethnicities=ref)* | - | - | - | - | - | - | - | - |
| White-British | 0.72 | (0.44, 1.19) | 0.05 | (-0.32, 0.43) | 0.68 | (0.41, 1.13) | 0.05 | (-0.32, 0.42) |
| *Long-standing illness/disability (yes=ref)* | - | - | - | - | - | - | - | - |
| No | 0.63 | (0.40, 1.35) | 0.65 | (0.31, 0.99) | 0.62 | (0.39, 0.98) | 0.65 | (0.31, 0.99) |
| *Income (less than £15,000=ref)* | - | - | - | - | - | - | - | - |
| £15,000-£25,000 | 0.60 | (0.32, 1.11) | -0.20 | (-0.65, 0.25) | 0.66 | (0.35, 1.22) | -0.19 | (-0.64, 0.26) |
| £25,000-£35,000 | 0.74 | (0.41, 1.35) | 0.07 | (-0.37, 0.52) | 0.81 | (0.44, 1.49) | 0.08 | (-0.36, 0.53) |
| £35,000-£50,000 | 0.75 | (0.39, 1.41) | 0.13 | (-0.35, 0.60) | 0.75 | (0.39, 1.43) | 0.13 | (-0.35, 0.60) |
| £50,000 and above | 1.04 | (0.57, 1.92) | 0.62 | (0.16, 1.07) | 1.09 | (0.58, 2.02) | 0.62 | (0.16, 1.08) |
| Don't know | 0.60 | (0.27, 1.31) | -0.35 | (-0.96, 0.23) | 0.63 | (0.28, 1.40) | -0.34 | (-0.92, 0.23) |
| *Long-term nature visit propensity (less frequent visitors=ref)* | - | - | - | - | - | - | - | - |
| More frequent visitors | 1.22 | (0.79, 1.86) | 0.21 | (-0.11, 0.52) | 1.20 | (0.78, 1.85) | 0.20 | (-0.11, 0.52) |
| *Short-term visit propensity* | 1.00 | (0.97, 1.04) | 0.02 | (-0.01, 0.05) | 1.01 | (0.97, 1.05) | 0.02 | (-0.01, 0.05) |
| N.B To aid interpretation, in the model with an interaction between the experimental brochure condition and recreational walking status, the reference category effectively becomes "non-walkers" who read the original brochure, odds ratios and coefficients for the enhanced brochure represent "non-walkers" who read the enhanced brochure, odds ratios and coefficients for "walker" represent "walkers" who read the original brochure, and the interaction term represents "walkers" who read the enhanced brochure. Short-term visit propensity is entered into regressions as a continuous predictor. | | | | | | | | |

Supplementary Figure A. Mediation model for “walkers”.


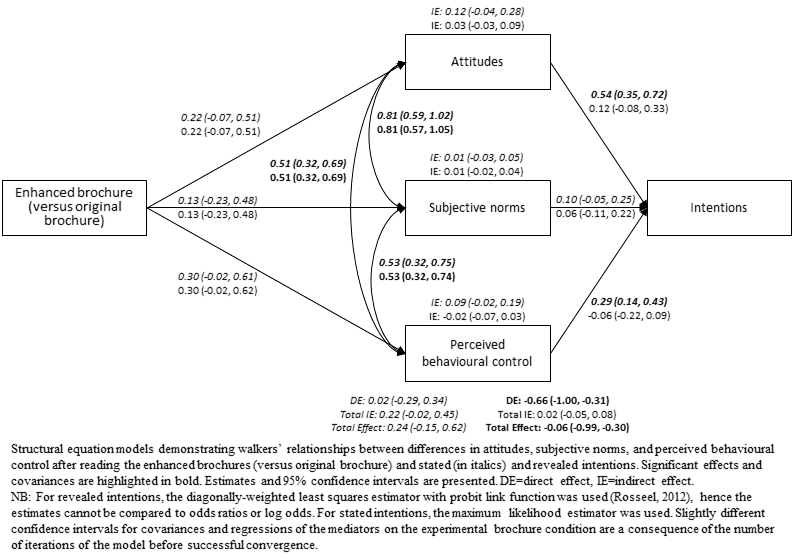


Supplementary Text A. Online questionnaire as it appeared in the experiment

**Perceptions of Outdoor Walking Routes**

**Information Sheet for Participants**

In this study, we are interested in what you think about outdoor recreational walking routes.

You’ll be asked to answer a series of background questions about yourself, then you will be given some information about outdoor recreational walking routes, and lastly you will answer a series of questions on your perceptions of these routes. **Please try and answer all questions as fully and honestly as possible.**

**To continue, please tick the boxes to confirm that:**

- **you** have read and understand the requirements for this study


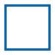


- **you** understand any information given by you may be used in future reports, articles or presentations by the research team.


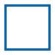


- **you** understand that your participation is voluntary and that you are free to withdraw at any time by closing your browser, without giving any reason.


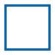


Many thanks

*ANONYMISED FOR PEER-REVIEW* (principal investigator)

**Perceptions of Outdoor Walking Routes**

**Please do not use the ‘back’ button of your browser at any time. Make sure you read the information on each page in full before continuing.**

When we refer to “walking routes” we mean walking through trails/routes/paths for pleasure in outdoor natural environments such as open spaces in and around towns and cities, the coast and the countryside.

These could be short or long, circular or linear, easy or difficult, marked or unmarked, walking routes. We do not mean walking these routes for transport purposes (to get to and from places) or walking around your own private garden or land.

**Q. Please check the statement that most applies to yourself:**


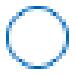
 I do not regularly complete walking routes and am not thinking about starting


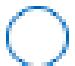
 I do not regularly complete walking routes but I am thinking about starting (but not in the next month)


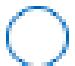
 I do not regularly complete walking routes but am thinking about starting in the next month


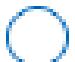
 I do regularly complete walking routes and have done so for less than 6 months
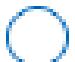
 I do regularly complete walking routes and have done so for over 6 months

**Leisure Time Out of Doors**

**We would like to know when you made out of doors visits in the last two weeks.**

By out of doors we mean open spaces in and around towns and cities, the coast and the countryside. This could be anything from a few minutes to all day. It may include time spent close to your home, further afield or while on holiday. However this does not include routine shopping trips or time spent in your own garden.

**Q. How often did you make this type of visit in the last two weeks?**

*(Please write in number)*

**Leisure Time Out of Doors**

**Q. Thinking about the last 12 months, how often, on average, have you spent your leisure time out of doors, away from your home?**

Again, by out of doors we mean open spaces in and around towns and cities, the coast and the countryside.


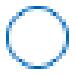
 More than once per day


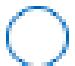
 Every day


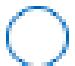
 Several times a week


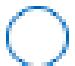
 Once a week


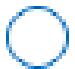
 Once or twice a month


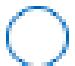
 Once every 2-3 months


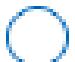
 Once or twice
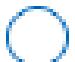
 Never

**About you**

**Q. Which of these best describes your ethnic group?**


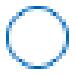
 **White:**English / Welsh / Scottish / Northern Irish / British


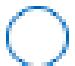
 Irish


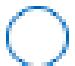
 Gypsy or Irish Traveller


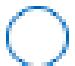
 Any other White background


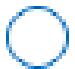
 **Mixed / multiple ethnic groups:**White and Black Caribbean


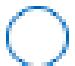
 White and Black African


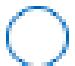
 White and Asian


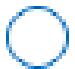
 Any other Mixed / multiple ethnic background


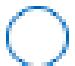
 **Asian / Asian British:**Indian


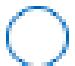
 Pakistani


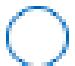
 Bangladeshi


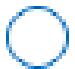
 Chinese


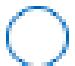
 Any other Asian background


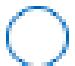
 **Black / African / Caribbean / Black British:**African


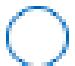
 Caribbean


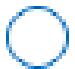
 Any other Black / African / Caribbean background


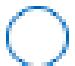
 **Other ethnic groups:**Arab


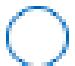
 Other not listed


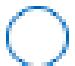
 **Prefer not to answer**

**About you**

**Q. Do you have any long standing illness, health problem or disability that limits your daily activities or the kind of work you can do?**


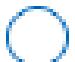
 Yes
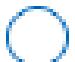
 No

**About you**

**Q. Which of the following best represents your total household income per year (before tax)?**


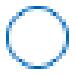
 Less than £15,000


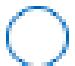
 £15,000 to £24,999


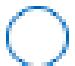
 £25,000 to £34,999


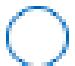
 £35,000 to £49,999


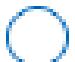
 £50,000 or more
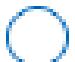
 Don't know

**Read the Leaflet**

**Please now read the following leaflet about an outdoor recreational walking route.**

As you read the leaflet, try to imagine yourself on this walk. Pay attention to the colours. Notice the textures. Imagine yourself breathing in the air; notice any smells that may be present. Think carefully about the details before proceeding.

It's really important that you read the whole leaflet from start to finish and understand what the leaflet is saying so that you can answer the questions in the next section. Reading the leaflet should take around 4 to 5 minutes.

(Click the image to open the leaflet as a larger image in a new tab in your browser. Once opened, use the magnifying glass cursor to zoom in or use the zoom function in your browser.)


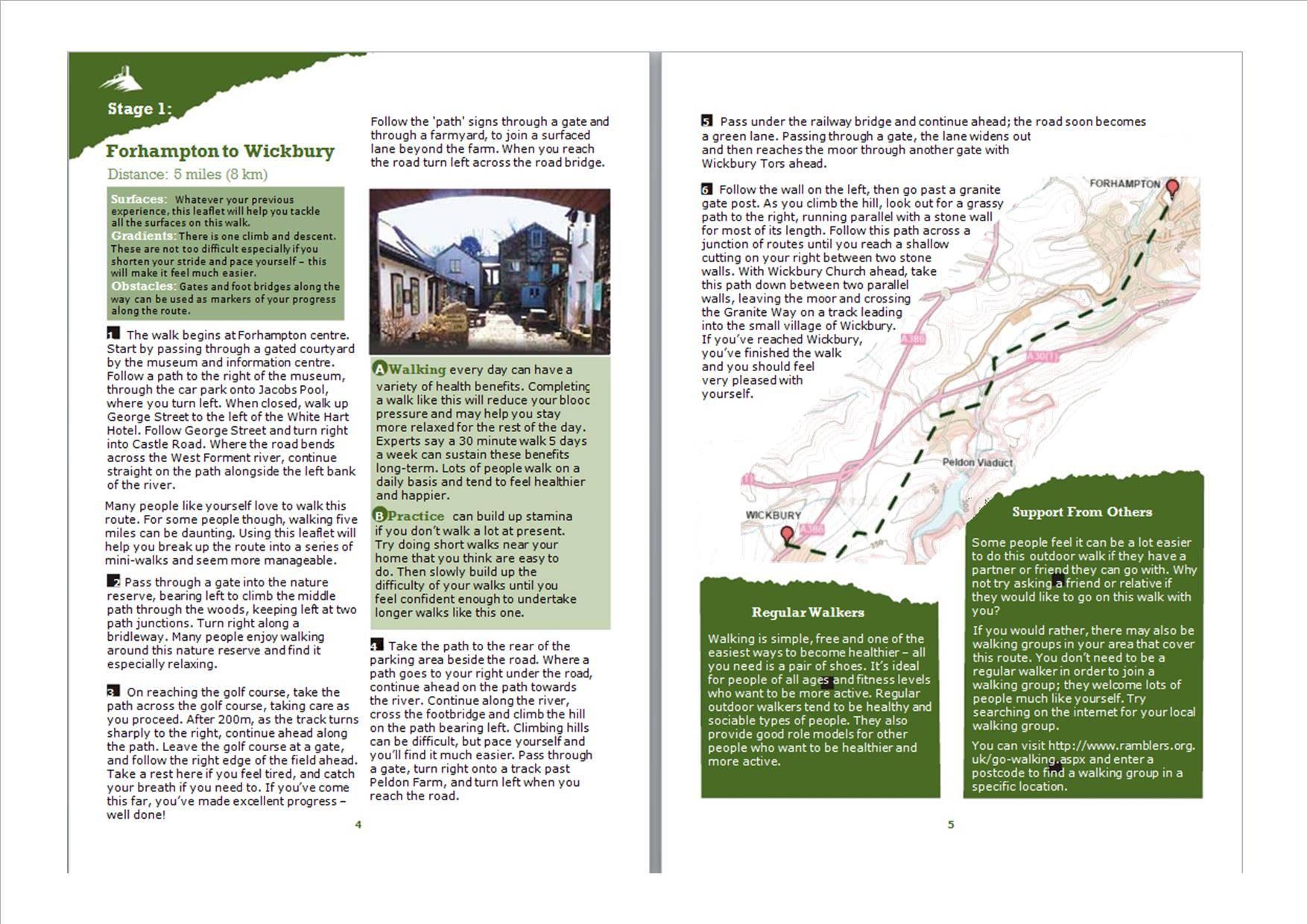


**Q. Please confirm that you have read the leaflet fully:**


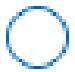
 **Yes** - I have read the leaflet fully


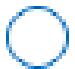
 **No** - I have not read the leaflet (and I wish to leave the study)

**Perceptions of Outdoor Walking Routes**

**Q. In light of what you have just read, please answer the following questions:**

**Bad**1 2 3 4 5 6 **Good**7

“Doing this outdoor walking route would be…”


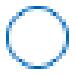

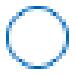

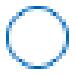

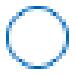

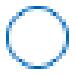

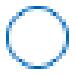

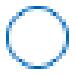


| **Very wo** |  |  |  |  |  | **Not at** |
| --- | --- | --- | --- | --- | --- | --- |
| **rthwhile** |  |  |  |  |  | **all wort** |
| 1 | 2 | 3 | 4 | 5 | 6 | **hwhile**7 |

"Doing this outdoor walking route would be…”


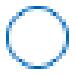

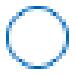

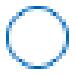

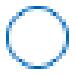

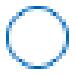

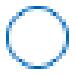

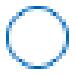


| **Pleasant** |  |  |  |  |  | **Unpleas** |
| --- | --- | --- | --- | --- | --- | --- |
| 1 | 2 | 3 | 4 | 5 | 6 | **ant**7 |

“Doing this outdoor walking route would be…”


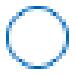

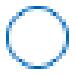

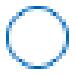

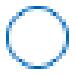

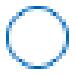

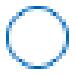

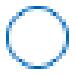


**Exciting**1 2 3 4 5 6 **Boring**7

“Doing this outdoor walking route would be…”


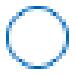

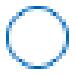

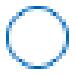

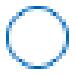

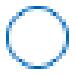

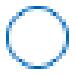

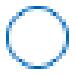


**Perceptions of Outdoor Walking Routes**

**Q. Please answer the following questions:**

|  |  |  |  |  |  | **Strongly** |
| --- | --- | --- | --- | --- | --- | --- |
| **Strongly** |  |  |  |  |  | **disagree** |
| **agree**1 | 2 | 3 | 4 | 5 | 6 | 7 |

“Other people like me complete walking routes like this.”


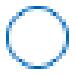

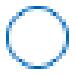

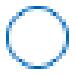

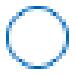

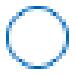

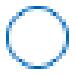

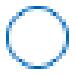


|  |  |  |  |  |  | **Strongly** |
| --- | --- | --- | --- | --- | --- | --- |
| **Strongly** |  |  |  |  |  | **disagree** |
| **agree**1 | 2 | 3 | 4 | 5 | 6 | 7 |

“A wide range of people complete walking routes like this.”


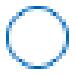

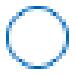

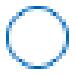

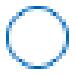

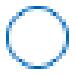

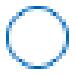

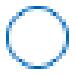


**Perceptions of Outdoor Walking Routes**

**Q. Please answer the following questions:**

**True**1 2 3 4 5 6 **False**7

“I am confident I could complete walking routes like this.”


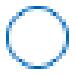

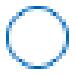

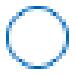

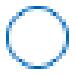

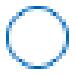

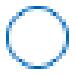

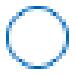


| **Very** |  |  |  |  |  | **Very** |
| --- | --- | --- | --- | --- | --- | --- |
| **difficult**1 | 2 | 3 | 4 | 5 | 6 | **easy**7 |

“Completing walking routes like this would be…”


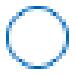

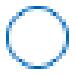

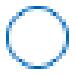

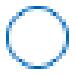


|  |  |  |  |  |  | **Strongly** |
| --- | --- | --- | --- | --- | --- | --- |
| **Strongly** |  |  |  |  |  | **disagree** |
| **agree**1 | 2 | 3 | 4 | 5 | 6 | 7 |

“I would be willing to complete walking routes like this.”

|  |  |  |  |  |  | **Strongly** |
| --- | --- | --- | --- | --- | --- | --- |
| **Strongly** |  |  |  |  |  | **disagree** |
| **agree**1 | 2 | 3 | 4 | 5 | 6 | 7 |

“In the future, I intend to complete walking routes like this.”

**Perceptions of Outdoor Walking Routes**

**Q. If you feel that reading the leaflet somehow changed your motivation to go on outdoor recreational walks, please explain why here.**

If possible, give examples from the leaflet that you can remember that made you feel this way.

**Thank you for your participation**

**If you would like further information on walking in your area, please select “show me walking information and submit” below. If you do not wish to be shown this, please just select “take me to submission page.”**

Show me walking information and submit

Take me to submission page

**Thank you**

Sorry you do not want to continue with the study. To let you know what is was about; we were mainly interested in how two versions of the same leaflet influenced your intentions to do outdoor recreational walks in the future. One had text designed to be especially persuasive whereas one was an original leaflet. If you have any questions regarding the study, please contact *ANONYMISED FOR PEER-REVIEW* on *ANONYMISED FOR PEER-REVIEW*.

**You may now close your web browser.**

**Thank you ... almost complete**

Thanks. This study has been conducted for a doctoral research project at the *ANONYMISED FOR PEER REVIEW*.

In this study, you were allocated to one of three groups. One group read an original outdoor recreational walking leaflet where we had replaced the place names with imaginary ones. The second read a "high quality" one where we modified the text to include information that we thought would be more persuasive than the original. The third group did not see either brochure. The idea behind doing this is to show that a "high quality" leaflet would result in more favourable attitudes and intentions towards outdoor recreational walking, but also that the original leaflet is better at improving attitudes and intentions than no information at all.

On the previous page we asked whether you would like to see more information in your local area – we predicted that you would be most likely to select this option if you had read the “high quality” leaflet.

If you do wish to get more information on walking in your local area, search for “The Rambler’s” website after you have pressed “submit”. Using this you can find guided and unguided walks near where you live.

Please email *ANONYMISED FOR PEER-REVIEW* on [*ANONYMISED](mailto:L.Elliott@exeter.ac.uk) FOR PEER-REVIEW* if you require any further information or wish to know the results of the study when they are published.

>> PLEASE NOW CLICK **SUBMIT** TO REGISTER YOUR PARTICIPATION IN THE SURVEY.

Supplementary Text B. Details, description, and results pertaining to the third experimental condition.

The following pages detail a third experimental condition that was originally included in the study but omitted from the main manuscript as it did not relate to our central aim of informing the future design of outdoor recreational walking brochures for different target demographics. Readers are welcome to contact the corresponding author for data and analysis scripts pertaining to this third condition.

*Method*

Respondents in this condition viewed no brochure in order to ascertain intentions for walking in natural environments in the absence of any reading material. All measures were the same as in the other two conditions. The procedure was also identical apart from that these participants were asked to do the following:

“We would now like you to imagine an outdoor recreational walking route. Pay attention to the colours. Notice the textures. Imagine yourself breathing in the air; notice any smells that may be present. Shut your eyes for a minute or two and imagine yourself on this walk. Think carefully about the details before proceeding as we will ask you some questions about the walk in the next section. When you're ready, click next to continue...”

This text was chosen to maximise the likelihood that participants in this condition would spend an amount of time imagining the walk that was comparable to the time needed to read brochures.

The analysis strategy was also identical but we omit additional structural equation models as it was only ever our intention to demonstrate that the two brochure conditions influenced antecedents of intentions formation differently.

*Results*

As a function of including this condition in analysis, but keeping the same exclusion criteria as we proposed in the analysis strategy, the numbers of participants excluded from each condition are different to the numbers present in the article where only two conditions are presented. For transparency, the results presented here display comparisons between all three experimental conditions.

Initially, 788 participants were recruited (no-brochure n=253, original n=269; enhanced n=266). Similar to the main article, 22 participants were initially excluded for indicating that they had not read the brochure in full (8=original, 14=enhanced), 5 did not have their sex or age successfully logged by the online platform, and 186 completed the experiment in under three minutes. At this stage, participants had a mean completion time of 8.6 minutes with a standard deviation of 15.5 minutes. Therefore, any participants completing the experiment in over 24.1 minutes were also excluded. This resulted in a further 18 exclusions and a resultant sample size of 557 (no brochure=155, original=205, enhanced=197).

Females made up 55% of the sample and the mean age was 42. Respondents were not excluded differentially by recreational walking status. Before exclusions, 417 respondents (53%) were classified as walkers and 371 (47%) as non-walkers. Afterwards, 300 (54%) were classified as walkers and 257 (46%) as non-walkers. There were no differences between the three conditions in terms of age (F(2, 554)=0.01, p=.99, ηp=.00), gender (X2(2, N=557)=0.12, p=.94), ethnicity (X2(2, N=557)=0.38, p=.83), household income (X2(10, N=557)=11.48, p=.32) disability status (X2(2, N=557)=0.85, p=.66), propensity to visit natural environments in the last two weeks (F(2, 554)=0.01, p=.99, ηp=.00), propensity to visit natural environments in the last 12 months (X2(14, N=557)=12.39, p=.58), or recreational walking status (X2(2, N=557)=1.18, p=.55). Descriptive statistics are displayed in Table B1 below.

*Did the enhanced brochure strengthen intentions to walk for recreation in natural environments?*

Overall neither the enhanced brochure (OR=0.81, 95% CI: 0.53, 1.21) nor the no-brochure condition (OR=0.93, 95% CI: 0.60, 1.44) resulted in higher revealed intentions than reading the original brochure. However, people classified as walkers did have stronger revealed intentions than non-walkers (OR=1.59, 95% CI: 1.10, 2.32). Associations with covariates were similar to the main article, but in addition, participants classified as white-British had significantly lower revealed intentions than participants classified as all other ethnicities (OR=0.59, 95% CI: 0.36, 0.95).

The enhanced brochure did result in significantly higher stated intentions than the original brochure (b=0.33, 95% CI: 0.03, 0.62), whilst reading no brochure resulted in marginally higher stated intentions (b=0.30, 95% CI: -0.01, 0.62). Again, walkers had higher stated intentions than non-walkers (b=1.08, 95% CI: 0.81, 1.34). For this outcome, significant associations with sociodemographic covariates were comparable to the main article.

*Was the effect of the enhanced brochure on intentions stronger for "non-walkers"?*

After adjusting for the interaction between the experimental brochure conditions and recreational walking status, a distinct pattern of results emerged. As predicted, for non-walkers, reading the enhanced brochure resulted in significantly higher revealed intentions (OR=2.34, 95% CI: 1.24, 4.53) compared to reading the original brochure, but surprisingly so did reading no brochure at all (OR=2.41, 95% CI: 1.21, 4.89). As expected, walkers reading the original brochure had higher revealed intentions than non-walkers reading the original brochure (OR=5.00, 95% CI: 2.67, 9.64), but walkers reading the enhanced brochure had substantially lower revealed intentions than non-walkers reading the original brochure (OR=0.15, 95% CI: 0.06, 0.35). Additionally, walkers reading no brochure at all also had significantly lower revealed intentions than non-walkers reading the original brochure (OR=0.20, 95% CI: 0.08, 0.49). These results are summarised in Table B2 below.

Non-walkers who read the enhanced brochure also had significantly higher stated intentions than non-walkers who read the original brochure (b=0.48, 95% CI: 0.04, 0.91) while non-walkers reading no brochure had marginally higher stated intentions (b=0.42, 95% CI: -0.05, 0.89). As expected, walkers who read the original brochure had markedly higher stated intentions that non-walkers who read the original brochure (b=1.23, 95% CI: 0.81, 1.66). Unlike revealed intentions, walkers reading either the enhanced brochure (b=-0.28, 95% CI: -0.87, 0.32) or no brochure at all (b=0.21, 95% CI: -0.84, 0.42) had only non-significantly lower stated intentions than non-walkers who read the enhanced brochure. This interaction is displayed graphically in Figure B1.

For comparability with the main article, we re-ran regressions with previously excluded individuals included in analysis (Online Appendix F contains the full analysis script pertaining to this analysis). In line with the contention in the main manuscript (that excluded participants were not paying sufficient attention to the task), all effects were weakened, rendering some previously significant effects insignificant. In models unadjusted for the interaction between the experimental conditions and recreational walking status, the effect of the enhanced brochure on stated intentions became insignificantly higher than the original brochure (b=0.22, 95% CI: -0.05, 0.49) which remained after adjustment for the interaction (b=0.33, 95% CI: -0.07, 0.72). Additionally, non-walkers who read no brochure now had non-significantly higher revealed intentions than non-walkers who read the original brochure (OR=1.64, 95% CI: 0.93, 2.90).

*Discussion*

In the main manuscript, all discussion points made regarding the two key conditions still hold true for this three-condition comparison. Here, we focus solely on the effects of this third no-brochure condition. For non-walkers, reading no brochure at all seemed to raise intentions of a similar magnitude to reading the enhanced brochure. One possible explanation concerns differential exclusion of participants by condition. Firstly, ninety-eight were excluded from the no brochure condition compared to 64 and 69 from the original and enhanced brochure conditions respectively. The task given to respondents with no brochure was to close their eyes and imagine a walking route for a minute or two. It is likely that the respondents excluded from the no brochure condition due to completing the survey too quickly either did not, or could not, imagine such a walking route. Therefore, the respondents remaining in the no brochure group were likely to be individuals who visualised an ideal walking route, which would, perhaps misguidedly, strengthen intentions as they imagine a high likelihood of successfully completing such walking routes (Oettingen, 2012). For example, the walk that included participants might have imagined would likely have been one that they do regularly, or that they have more positive attitudes towards, that more peers complete, or that they have higher confidence for completing. Therefore, it could be because of the exclusion criteria that non-walkers’ reported intentions after reading no brochure were higher than after reading the standard brochure.

Conversely, for people classified as walkers, this imagination task appeared to decrease the likelihood that they would request further walking information at the end of the study (see Figure 1 below). Previous literature has claimed that walking brochures are typically designed to appeal to experienced walkers (Elliott et al., 2016), and the main article's findings show that a typical brochure is persuasive for this subgroup while an enhanced brochure is dissuasive. This additional data shows that even compared to reading no information at all, a typical walking brochure still significantly heightens the intentions of more experienced walkers.

*Implications*

One could conclude that providing tailored, theory-based information on a recreational walking route through natural environments is approximately as effective at raising walking intentions for non-walkers as giving them the time to imagine a walking route. Unlike the implementation of new brochure guidelines and recreational walking brochures, we are unsure of how such rumination on walks could be facilitated in reality as an effective intervention. In any case, people who do take the time to imagine such walks, may then seek out written information to facilitate them performing similar walks anyway, and thus the analyses in the main article applies. It is for these reasons that the analyses presented here are excluded from the main manuscript, but we retain them here for full transparency.

*References*

Elliott, L. R., White, M. P., Taylor, A. H., & Abraham, C. (2016). How do brochures encourage walking in natural environments in the UK? A content analysis. Health Promotion International. DOI: 10.1093/heapro/daw083.

Oettingen, G. (2012). Future thought and behaviour change. European Review of Social Psychology, 23(1), 1-63.

| Table B1. Descriptive statistics for the two outcome variables and three proposed mediator variables. | | | | | | | | | | |
| --- | --- | --- | --- | --- | --- | --- | --- | --- | --- | --- |
|  |  | Overall (n=557) | | | Non-walkers (n=257) | | | Walkers (n=300) | | |
|  |  | No-brochure (n=155) | Original brochure (n=205) | Enhanced brochure (n=197) | No-leaflet (n=69) | Original brochure (n=91) | Enhanced brochure (n=97) | No-leaflet (n=86) | Original brochure (n=114) | Enhanced brochure (n=100) |
| Revealed intentions | *% SE* | 41.29 3.95 | 43.90 3.47 | 39.09 3.48 | 42.03 5.94 | 23.08 4.42 | 42.47 5.02 | 40.70 5.30 | 60.53 4.58 | 36.00 4.80 |
| Stated intentions | *M SD* | 5.35 1.68 | 4.97 1.77 | 5.30 1.55 | 4.67 1.83 | 4.19 1.67 | 4.74 1.63 | 5.90 1.32 | 5.60 1.59 | 5.85 1.24 |
| Attitudes | *M SD* | 5.58 1.06 | 5.26 1.18 | 5.50 1.27 | 5.41 1.08 | 4.85 1.21 | 5.19 1.37 | 5.72 1.03 | 5.58 1.05 | 5.81 1.08 |
| Normative beliefs | *M SD* | 5.16 1.42 | 5.15 1.39 | 5.32 1.46 | 5.11 1.34 | 4.67 1.36 | 4.96 1.52 | 5.21 1.50 | 5.53 1.30 | 5.67 1.32 |
| Self-efficacy | *M SD* | 5.16 1.52 | 4.95 1.51 | 5.30 1.47 | 4.71 1.84 | 4.34 1.64 | 4.84 1.62 | 5.52 1.09 | 5.43 1.21 | 5.75 1.15 |
| N.B Mean self-reported intention scores represent the average of two 7-point rating scales which were recoded such that 1=strongly disagree and 7=strongly agree. Mean attitude score comprised the average score of four 7-point attitudinal items see 2.3.3. Mean descriptive norm score and mean self-efficacy score comprised the average of two 7-point items each (see 2.3.3). Recreational walking status was dichotomised into two groups representing those who self-reported being in the precontemplation, contemplation, and preparation stages of change (“non-walkers”) and those who self-reported being in the action and maintenance stages of change (“walkers”). | | | | | | | | | | |

| Table B2. Fully-adjusted odds ratios and regression coefficients, together with 95% confidence intervals, for models which predict revealed and stated intentions. Models both without and with an interaction between experimental brochure condition and recreational walking status are presented (hypotheses (a) and (b) respectively). | | | | | | | | |
| --- | --- | --- | --- | --- | --- | --- | --- | --- |
|  | Models without interaction | | | | Models with interaction | | | |
|  | Revealed intentions | | Stated intentions | | Revealed intentions | | Stated intentions | |
| *Fully-adjusted (n=557)* | (Hosmer & Lemeshow=.04) (Nagelkerke=.07) | | (Adjusted R^2^=.20) | | (Hosmer & Lemeshow=.07) (Nagelkerke=.12) | | (Adjusted R^2^=.20) | |
|  | OR | CI | *b* | CI | OR | CI | *b* | CI |
| Intercept | 0.79 | (0.35, 1.78) | 3.56 | (2.97, 4.15) | 0.42 | (0.17, 1.02) | 3.48 | (2.86, 4.09) |
|  |  |  |  |  |  |  |  |  |
| *Brochure (original brochure=ref)* | - | - | - | - | - | - | - | - |
| No brochure | 0.93 | (0.60, 1.44) | 0.30 | (-0.01, 0.62) | 2.41 | (1.21, 4.89) | 0.42 | (-0.05, 0.89) |
| Enhanced brochure | 0.81 | (0.53, 1.21) | 0.33 | (0.03, 0.62) | 2.34 | (1.24, 4.53) | 0.48 | (0.04, 0.91) |
| *Recreational walking status ("non-walkers"=ref)* | - | - | - | - | - | - | - | - |
| "Walkers" | 1.59 | (1.10, 2.32) | 1.08 | (0.81, 1.34) | 5.00 | (2.67. 9.64) | 1.23 | (0.81, 1.66) |
| No brochure x "Walkers" | - | - | - | - | 0.20 | (0.08, 0.49) | -0.21 | (-0.84, 0.42) |
| Enhanced brochure x "Walkers" | - | - | - | - | 0.15 | (0.06, 0.35) | -0.28 | (-0.87, 0.32) |
|  |  |  |  |  |  |  |  |  |
| *Sex (male=ref)* | - | - | - | - | - | - | - | - |
| Female | 1.32 | (0.92, 1.89) | -0.05 | (-0.31, 0.20) | 1.24 | (0.86, 1.79) | -0.06 | (-0.32, 0.19) |
| *Age (18-34 year olds=ref)* | - | - | - | - | - | - | - | - |
| 35-48 year olds | 1.74 | (1.12, 2.72) | 0.08 | (-0.24, 0.39) | 1.73 | (1.10, 2.72) | 0.07 | (-0.24, 0.39) |
| 49-65 year olds | 1.39 | (0.89, 2.18) | -0.07 | (-0.39, 0.24) | 1.37 | (0.87, 2.17) | -0.08 | (-0.39, 0.24) |
| *Ethnicity (all other ethnicities=ref)* | - | - | - | - | - | - | - | - |
| White-British | 0.59 | (0.36, 0.95) | -0.06 | (-0.41, 0.29) | 0.57 | (0.35, 0.92) | -0.07 | (-0.42, 0.28) |
| *Long-standing illness/disability (yes=ref)* | - | - | - | - | - | - | - | - |
| No | 0.84 | (0.54, 1.31) | 0.71 | (0.39, 1.02) | 0.83 | (0.53, 1.30) | 0.71 | (0.39, 1.02) |
| *Income (less than £15,000=ref)* | - | - | - | - | - | - | - | - |
| £15,000-£25,000 | 0.55 | (0.30, 1.01) | 0.24 | (-0.19, 0.67) | 0.58 | (0.31, 1.07) | 0.25 | (-0.18, 0.68) |
| £25,000-£35,000 | 0.72 | (0.40, 1.29) | 0.19 | (-0.23, 0.62) | 0.75 | (0.41, 1.36) | 0.20 | (-0.22, 0.62) |
| £35,000-£50,000 | 0.66 | (0.35, 1.21) | 0.31 | (-0.14, 0.75) | 0.67 | (0.36, 1.25) | 0.31 | (-0.14, 0.76) |
| £50,000 and above | 0.96 | (0.54, 1.73) | 0.58 | (0.15, 1.01) | 0.96 | (0.53, 1.76) | 0.58 | (0.15, 1.01) |
| Don't know | 0.88 | (0.40, 1.93) | -0.36 | (-0.93, 0.21) | 0.95 | (0.42, 2.14) | -0.35 | (-0.92, 0.23) |
| *Long-term nature visit propensity (less frequent visitors=ref)* | - | - | - | - | - | - | - | - |
| More frequent visitors | 1.21 | (0.81, 1.82) | 0.12 | (-0.17, 0.41) | 1.21 | (0.80, 1.83) | 0.12 | (-0.17, 0.41) |
| *Short-term visit propensity* | 1.00 | (0.96, 1.04) | 0.02 | (-0.01, 0.05) | 1.00 | (0.96, 1.05) | 0.02 | (-0.01, 0.05) |
| N.B To aid interpretation, in the model with an interaction between the experimental brochure condition and recreational walking status, the reference category effectively becomes "non-walkers" who read the original brochure, odds ratios and coefficients for no brochure and the enhanced brochure represent "non-walkers" who read no brochure and the enhanced brochure respectively. Odds ratios and coefficients for "walker" represent "walkers" who read the original brochure, and the interaction terms represent "walkers" who read no brochure and the enhanced brochure. Short-term visit propensity is entered into regressions as a continuous predictor. | | | | | | | | |

Figure B1. The brochure condition by walking status interaction (both intention outcomes).
